# Recurrent deep vein thrombosis following brown recluse spider bite complicated by medication noncompliance and residual scar tissue: A rare case report

**DOI:** 10.1002/ccr3.7263

**Published:** 2023-04-25

**Authors:** Arihant Surana, Ayush Anand, Mohammaed Hazique, Birgurman Singh, Ashwini Gupta, John Georgy, Amir Kaki

**Affiliations:** ^1^ Nilratan Sircar Medical College and Hospital Kolkata India; ^2^ BP Koirala Institute of Health Sciences Dharan Nepal; ^3^ Government Medical College Patiala India; ^4^ Kasr Al‐Aini, Cairo Medical School Cairo Egypt; ^5^ School of Medicine Wayne State University Detroit USA

**Keywords:** brown recluse spider, case report, deep vein thrombosis, spider bite

## Abstract

Clinicians should be aware of the occurrence of deep vein thrombosis following brown recluse spider bite.

## 1 INTRODUCTION

Among the 11 Loxosceles species found in the USA, the brown recluse spider accounts for the majority of envenomation cases.^1^ Brown recluse spider is commonly found in the southeast part of the United States of America (USA) and is rarely found in the Michigan area.^1^, ^2^, ^3^ This spider is usually nonaggressive and bites only when provoked.^2^, ^3^ Though most of the bites are asymptomatic, rarely there can be severe cutaneous, hemolytic, and systemic manifestations.^1^, ^3^, ^4^, ^5^, ^6^, ^7^, ^8^, ^9^, ^10^, ^11^, ^12^ The diagnosis is done based on clinical evaluation coupled with the identification of species.^1^ So far, no case of deep vein thrombosis has been reported following a brown recluse spider bite. Herein, we present the rare case of recurrent deep vein thrombosis (DVT) following a brown recluse spider bite in the Michigan area.

## 2 CASE PRESENTATION

A 45‐year‐old male from Michigan, USA, with a past medical history of asthma presented to the emergency department (ED) complaining of left calf pain and swelling with ulcers following a brown recluse spider bite. Initially, the patient deferred physician care due to insurance issues but presented later due to a sudden increase in pain and swelling with ulcers in the bite region for 4 h. He did not have any venous thromboembolic disease or surgical interventions in the past. The patient was under medication for asthma, which was under control. The patient has been an active smoker and alcohol consumer for the past 30 years. The family history was notable for AIDS and breast cancer. He denied any family history of thromboembolism, bleeding, or clotting disorders.

Initial vital signs showed blood pressure of 127/85 mm Hg, heart rate of 70 beats per minute, respiratory rate of 18 breaths per minute, and temperature of 37.4°C, with oxygen saturation of 96% on room air. On examination, there was swelling and tenderness with +2 pitting edema in the right calf. Extremity pulses were normal bilaterally. The rest of the systemic examinations were unremarkable. The modified Wells' score for deep vein thrombosis was three points. His initial investigations are mentioned in Table 1.

**TABLE 1:** Laboratory investigations of the patient.

Investigations	Results	Reference range
Hb (g/dL)	13.5	11–16
TLC (cells/mm^3^)	6300	4000–11,000
Platelets (cells/mm^3^)	1,94,000	1,50,000–4,50,000
PT (second)	10.3	10–13
INR	0.96	<1.1
APTT(second)	26.2	30–40
MCV (fL)	91.4	80–96
MCH (pg)	29.7	27–34
MCHC (g/dL)	32.5	32–36
RBC (million/mL)	4.55	1.5–4.5
Serum Sodium (mmoL/L)	137	135–150
Serum Potassium (mmoL/L)	4.3	3.5–5.1
Serum Chloride	103	98–107

Abbreviations: APTT, activated partial thromboplastin time; Hb, hemoglobin; HBsAg, Hepatitis B surface antigen; HCV, hepatitis C virus; HIV, human immunodeficiency virus; INR, international normalized ratio; MCH, mean corpuscular hemoglobin; MCHC, mean corpuscular hemoglobin concentration; MCV, mean corpuscular volume; PT, prothrombin time; RBC, red blood cell; TLC, total leucocyte count.

Based on these findings, a provisional diagnosis of DVT was made, and the patient was further evaluated with an ultrasound (USG) scan. USG scan revealed nonocclusive DVT in the left mid to distal femoral vein, popliteal vein, and one of the paired posterior tibial veins. We did a d‐dimer test to look for pulmonary embolism, which was normal. Laboratory investigations did not suggest any abnormality.

Supportive management was done with 250 mL of intravenous (IV) 0.9% normal saline and 4 mg IV morphine. Anticoagulation was initiated with IV heparin. Despite IV heparin drip, he complained of persistent swelling and pain in his leg, and he was scheduled for a balloon thrombectomy. After the thrombectomy, an 18 × 90 mm stent was placed in the right common iliac vein and right external iliac. The patient was hemodynamically stable, and his symptoms improved after the procedure. He was discharged from the hospital on aspirin 81 mg QD and clopidogrel 75 mg QD for 1 month, and apixaban 10 mg bid for 7 days, followed by 5 mg bid for 3 months.

The patient was noncompliant with medication and presented to the ED 4 days later with discoloration, mild swelling, and worsening left leg pain. He was given a dose of aspirin, clopidogrel, and apixaban. Lower extremity venous duplex revealed acute deep vein thrombosis involving the right common femoral, femoral, popliteal, and peroneal veins. Successful thrombectomy of right common iliac vein stent thrombosis, right external iliac vein thrombosis, and right femoral vein thrombosis was performed. The patient was advised to continue dual antiplatelet therapy (aspirin and clopidogrel), and resume anticoagulation with apixaban 10 mg b.i.d. for 7 days followed by 5 mg b.i.d.

**FIGURE 1:**
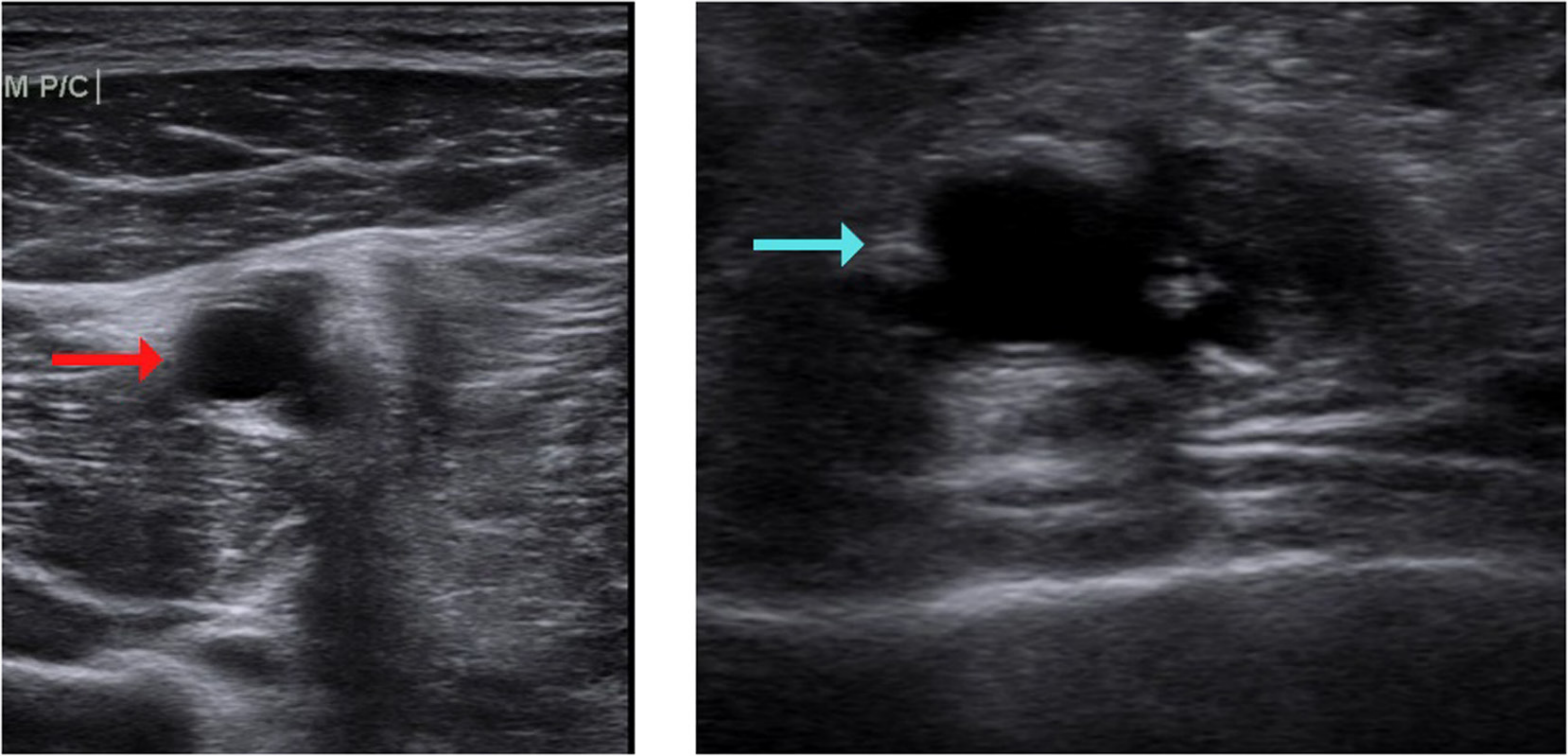
Lower extremity venous duplex showing revealed noncompressible right femoral vein (red arrow) and popliteal vein (blue arrow).

On his follow‐up visit a month later, he complained of persistent leg pain and leg swelling despite medication compliance. Lower extremity venous duplex (Figure 1) revealed right femoral vein and popliteal noncompressibility suggestive of recurrent DVT with positive venous insufficiency in the right great saphenous vein at the saphenofemoral junction. His right lower extremity venogram revealed chronic total occlusion of the right common femoral vein and saphenofemoral vein secondary to a chronic thrombus. Successful venoplasty of the right common femoral vein and the saphenofemoral vein was performed with recanalization of normal flow. The patient was asked to continue clopidogrel and apixaban and was scheduled for a follow‐up visit.

On his follow‐up visit, his symptoms persisted despite medication compliance. The lower extremity venous report revealed acute DVT in the right proximal to distal femoral vein, popliteal vein, and posterior tibial vein. Chronic DVT was noted in the right gastrocnemius vein and peroneal vein, right mid to distal femoral vein, popliteal vein, and gastrocnemius veins, along with superficial vein thrombosis in the right small saphenous vein. A right lower extremity venogram revealed an occluded femoral vein with multiple collaterals, a patent common femoral vein, and a patent common iliac stent. Multiple attempts to cross the occluded segment failed. The patient was asked to continue oral anticoagulation therapy with clopidogrel and was scheduled for a follow‐up visit 1 month later.

Despite medication compliance, the patient had an acute episode of leg pain with leg swelling and was admitted to the ER. A lower extremity venous duplex revealed chronic DVT involving the bilateral femoral and popliteal veins. The patient was started on an IV heparin drip, leading to symptomatic improvement. He was referred to a venous thromboembolism clinic, which suggested residual scar tissue serving as a nidus rather than an oral anticoagulation failure. He was advised to continue oral anticoagulants for life and continue antiplatelet therapy for a postiliac vein stent. The patient was encouraged to use compression stockings and start a compression pump for symptomatic relief. The patient was lost to follow‐up, so we could not obtain long‐term data.

## 3 DISCUSSION

Patients with DVT usually have asymmetric swelling, pain, and tenderness in the affected limb.^13^ The diagnosis is based on clinical evaluation and confirmation using an ultrasonography duplex scan.^13^ In our case, the duplex scan clearly showed incompressible veins, suggestive of DVT. We should look out for pulmonary embolism (PE) in these patients using Well's score.^13^, ^14^, ^15^ Patients with scores of 2–4 should undergo a d‐dimer test.^14^ Our patient had a moderate risk of pulmonary embolism for which a d‐dimer test was ordered, which came out to be normal. Hence, we did not order further investigations. In patients with DVT, anticoagulation therapy should be started. However, there is a significant risk of recurrence of DVT and post‐thrombotic syndrome.^16^, ^17^ To prevent the recurrence of DVT, anticoagulation along with thrombectomy should be done.^18^ Hence, we employed a similar approach with our patient. Due to noncompliance to oral anticoagulation therapy, the patient again presented with similar complaints. Again, we performed a thrombectomy of the affected veins, and counseling was done for medication adherence. On follow‐up, the patient had a recurrence of DVT. A duplex scan suggested chronic DVT, for which venoplasty was done.^19^ Despite this, the patient's condition did not improve much. Hence, the patient was sent to a venous thromboembolism clinic, which showed residual scar tissue. The large clot burden in venous system can lead to increased chances of residual venous thromboembolism and recurrent DVT.^20^ Hence, the cause of recurrence was due to the presence of residual scar tissue serving as a nidus rather than earlier thought oral anticoagulation failure. However, after this, the patient was lost to follow‐up, and further data could not be obtained. Also, the exact location of the scar could not be determined because of multiple failed anterograde and retrograde wiring attempts to cross the thrombosed segments.

Scarce data is available regarding the toxicological analysis of loxosceles species venom.^21^ Phospholipase D (PLD), a molecule found in venom, can lead to massive inflammation and platelet aggregation.^21^ Though establishing exact causation is difficult, we tried to rule out any risk factor which might have led to DVT.^3^, ^13^, ^14^, ^15^ In our patient, we did not find any other cause which might have contributed to DVT. Hence, PLD can be attributed to the development of deep venous thrombosis. This makes our case unique as, so far, no cases of DVT have been reported following a brown recluse spider bite.

## 4 CONCLUSION

Our case highlighted the rare occurrence of DVT following a brown recluse spider bite in a nonendemic area. We showed the approach to the evidence‐based management of DVT, which is similar to the usual approach to managing DVT. This case highlighted that clinicians should be aware about this uncommon occurrence.

## AUTHOR CONTRIBUTIONS

**Arihant Surana:** Conceptualization; validation; visualization; writing – original draft; writing – review and editing. **Ayush Anand:** Conceptualization; software; supervision; validation; visualization; writing – original draft; writing – review and editing. **Mohammad Hazique:** Writing – original draft; writing – review and editing. **Birgurman Singh:** Writing – original draft; writing – review and editing. **Ashwini Gupta:** Writing – original draft; writing – review and editing. **John Georgy:** Writing – original draft; writing – review and editing. **Amir Kaki:** Conceptualization; data curation; resources; supervision; validation; visualization; writing – original draft; writing – review and editing.

## FUNDING INFORMATION

The author(s) did not receive any funding for this work.

## CONFLICT OF INTEREST STATEMENT

The author(s) do not have any conflict of interests to declare.

## ETHICS STATEMENT

Ethical approval was not required for this case report.

## CONSENT

Written informed consent was obtained from the patient to publish this report in accordance with the journal's patient consent policy.

## Data Availability

This manuscript has all the data relevant to this case report included.
